# Retraction Note: Impacts of rice-husk biochar on soil microbial biomass and agronomic performances of tomato (Solanum lycopersicum L.)

**DOI:** 10.1038/s41598-025-14309-0

**Published:** 2025-10-07

**Authors:** Seun Owolabi Adebajo, Folasade Oluwatobi, Pius Olugbenga Akintokun, Abidemi Esther Ojo, Aderonke Kofoworola Akintokun, Ige Samuel Gbodope

**Affiliations:** 1 Department of Microbiology, Federal University of Agriculture, Abeokuta, Nigeria; 2 Department of Plant Physiology and Crop Production, Federal University of Agriculture, Abeokuta, Nigeria

Retraction of: *Scientific Reports* 10.1038/s41598-022-05757-z, published online 02 February 2022

Editors have retracted this Article.

After publication of this Article, concerns were raised about the tomato plant parameters (such as height, stem thickness, leaf surface area) for plants described in Figs. 3–7, as well as about the unexpectedly low number of flowers and low yields. Additionally, some of the parameters are reported with incorrect units. The Authors were asked to provide their original data but were unable to do so. Post-publication peer review confirmed the issues with the methodology and data. The Editor has therefore lost confidence in the results reported in this Article.

Seun Owolabi Adebajo disagrees with the retraction. Ige Samuel Gbodope did not explicitly state if they agree or disagree with this retraction. Folasade Oluwatobi, Pius Olugbenga Akintokun, Abidemi Esther Ojo, and Aderonke Kofoworola Akintokun did not respond to the correspondence from the Editors about the retraction.

